# Trauma research in low- and middle-income countries is urgently needed to strengthen the chain of survival

**DOI:** 10.1186/1757-7241-19-62

**Published:** 2011-10-24

**Authors:** Torben Wisborg, Thapelo R Montshiwa, Charles Mock

**Affiliations:** 1 Department of Acute Care, Hammerfest Hospital, Hammerfest, Norway; 2 Anaesthesia and Critical Care Research Group, Faculty of Health Sciences, University of Tromsø, 9037 Tromsø, Norway; 3 Orthopedics, Faculty of Health Sciences- School of Medicine, University of Botswana, Gaborone, Botswana; 4 Harborview Injury Prevention and Research Center, University of Washington, Seattle, Washington, USA

## Abstract

Trauma is a major - and increasing - cause of death, especially in low- and middle income countries. In all countries rural areas are especially hard hit, and the distribution of physicians is skewed towards cities. To reduce avoidable deaths from injury all links in the chain of survival after trauma needs strengthening. Prioritizing in each country should be done by local researchers, but little research on injuries emerges from low- and middle income countries. Researchers in these countries need support and collaboration from their peers in industrialized countries. This partnership will be of mutual benefice.

## 

Every day, 16,000 men, women, and children are killed by injuries, and thousands more are permanently injured worldwide. It is estimated that for every death there are dozens of hospital admissions, hundreds of emergency department visits, and thousands of doctors' appointments, in the countries where such facilities exists. Injuries are responsible for six of the 15 leading causes of death in 15 to 44 year-olds worldwide [1]. Without new or improved interventions, road traffic injuries will be the third leading cause of death worldwide by the year 2020 [2].

This deadly epidemic, devastating to all involved, is hitting victims with least resources. Almost 90% of deaths due to injuries occur in low- and middle-income countries (LMIC) [3]. Injuries from road traffic accidents, interpersonal violence, and war are among the leading causes of death in low- and middle-income countries [2].

The distribution of resources is skewed in these countries, with most physicians and medical facilities located in major cities [4]. The rate of prehospital death is highest in the countries with least resources [5]. Worldwide, there is a mismatch between the distribution of doctors and injuries (Figures 1 and 2) [6,7].

**Figure 1:**
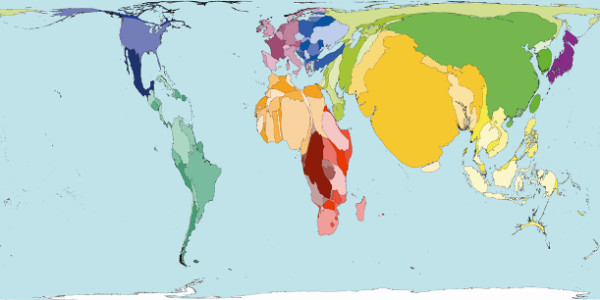
**Where is the problem? Territories are sized in proportion to the absolute number of people who died from injuries in 2002**. [6]

**Figure 2:**
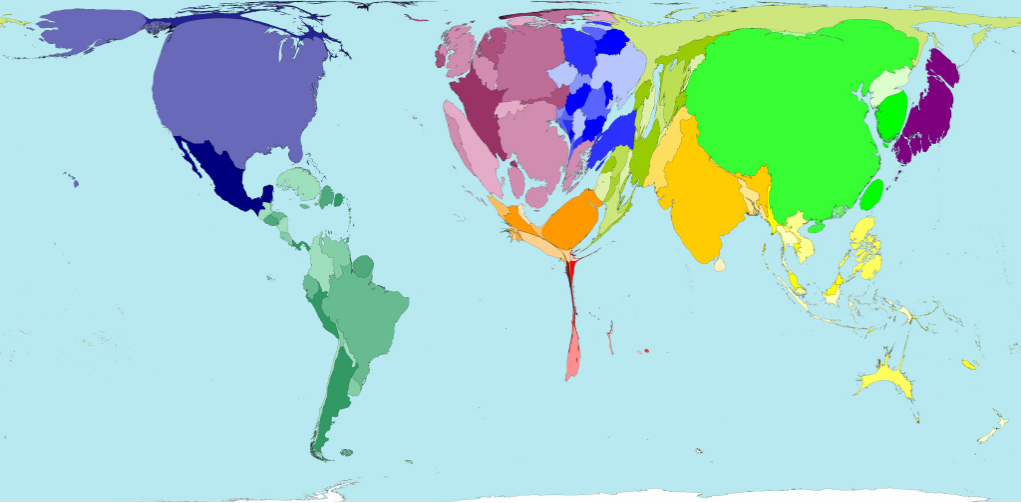
**Where are the doctors? Territory size shows the proportion of all physicians (doctors) that work in that territory (2004)**. [7]

In comparison to the high income countries little research is published from low- and middle income countries. Roy and co-workers state in a recent publication that "Considering that 85% of disasters and 95% of disaster-related deaths occur in the developing world, the overwhelming number of casualties has contributed insignificantly to the world's peer-reviewed literature. Less than 1% of all disaster-related publications are about disasters in the developing world [8]".

It is thus commendable that two groups of researchers from Tanzania [9] and Nigeria [10] are reporting their experiences in Scandinavian Journal of Trauma, Resuscitation and Emergency Medicine. Chalya and co-workers describe a situation in their referral level intensive care department with high burdens from injuries, mainly road traffic injuries [9]. These patients were severely injured, and the authors found a correlation between delay in ICU admission and mortality, amongst others. Iteke and co-workers have investigated the frequency of post-traumatic stress disorder (PTSD) in injury victims after road traffic injuries [10]. One of the findings was a correlation between lack of family income and risk of PTSD development. Both groups of researchers are thus in the more advanced end of the chain of survival.

These two articles provide us with very useful information that helps to further the field of trauma care globally, pointing out priorities both for action based on what we know already and for further research. In terms of action, Iteke and co-workers have pointed out the dearth of services available for the very large numbers of injury victims with PTSD. This fits closely with the sparseness of rehabilitation capabilities more broadly in many LMICs. For example, a survey of trauma care capabilities in 4 LMICs revealed that rehabilitation services such as physio-therapy and prostheses were very inadequate and the services of more highly specialized rehabilitative personnel were nearly completely absent in almost all circumstances evaluated, except for urban areas of middle income countries [11]. In general, such rehabilitative services were at a lower level of development and availability than were acute care services. The current study adds more evidence to these deficiencies and further shows that psychological aspects of rehabilitation need to be addressed as well. Increase in capabilities for physical and psychological rehabilitation of injury victims can thus be seen as priorities for action based on what we know already.

In terms of research priorities, Chalya and co-workers point out the large number of injury victims who need ICU care. Issues of priorities for ICU care in LMICs and related issues of what are the most cost-effective elements of ICU care that should be more widely promoted have been scarcely addressed in the world's literature. For example, what minimum core of procedures should be assured, what levels of staffing for which types of providers should be promoted, and what types of equipment and supplies should be stocked in ICUs globally are all questions that need to be answered before firm recommendations about ICU care globally can be made. These are clearly priorities for future research.

Inhabitants of rural areas, be it in low- and middle income countries or in high income countries, will often never reach these advanced treatment facilities. Prehospital mortality rates are above 70% in both rural Europe and LMIC [12,13]. Is it possible to strengthen the chain of survival even in the initial links? Yes, and several papers do in fact indicate this [14-16]. A recent study from Northern Iraq indicates that not only training of paramedics in a long-time project, but also short-time first responder training have a significant individual impact on mortality after injuries in rural areas of Northern Iraq [17]. These lower cost alternatives for prehospital care in LMICs have received limited attention compared with research on higher cost EMS systems from high income countries [18].

It is thus high time to use existing knowledge to bridge the gaps in trauma care in low- and middle income countries. The two recent papers in Scandinavian Journal of Trauma, Resuscitation and Emergency Medicine [9,10] are good examples of local researchers taking responsibility for their link of the trauma chain of survival. Several papers on prehospital and in-hospital care improvement underline the need for a systematic approach [19], based on a careful needs assessment, which in turn will direct the efforts towards areas with expected high return of investments. Local researchers are the key to this knowledge, and need to disseminate their experience to the international audience. It should, however, be acknowledged that the challenges faced by most local researchers in LMICs are great, and with limited resources, research is usually given one of the least of priorities. Support from the international community will therefore play a very important role, not just in funding but also from the expertise of other experienced and well published researchers in the developed countries and institutions. This valuable role can be in the form of collaborations, mentorship, guidance and other similar support, therefore generally promoting the culture of research and publication.
